# Lack of Interest in the Disease of Inebriety by the General Profession

**Published:** 1887-07

**Authors:** Edward C. Mann

**Affiliations:** No. 128 Park Place, near Prospect Park, Brooklyn, N. Y.


### DOMESTIC CORRESPONDENCE.

### LACK OF INTEREST IN THE DISEASE OF INEBRIETY BY THE GENERAL PROFESSION.

*To the Editor of the Chicago Medical Journal and Examiner :*

WHEN we reflect upon the fact that an insane temperament, which may be transmitted to offspring, may be established by the alcohol habit; that the alcoholic appetite may originate in moderate indulgence to become fixed and hereditary; that epilepsy, hysteria, idiocy and insanity result from the alcohol habit and become hereditary; that the people of the United States spent, in the period of eleven years, for alcohol more than the entire value of their agricultural, mechanical and manufacturing products; that 33⅓ per cent. of all deaths in New York, and probably a similar per cent. in every large city, are occasioned, directly or indirectly, by the use of alcoholic drink; that from 13 to 25 per cent. of all cases of insanity become insane through alcoholic drink; that the progeny of alcoholics are peculiarly liable to degenerations of the nervous system; that the alcoholic habit is antagonistic to the highest standard of bodily health and vigor and of capacity for work—then every thoughtful physician must realize that we have before us one of the deadliest evils that curses modern society, and we can but wonder at the astonishing apathy that exists in regard to the importance of a thorough knowledge of the disease of inebriety in the daily work of every member of the profession.

The chief study in this direction ought not to be limited to the very small band of specialists in the profession who come together at the annual meetings of the American Association for the Cure of Inebriates. Such an association should number its hundreds of members. The disease of inebriety does not chiefly fall, in its practical relations, within the working limits of this exceedingly small band of specialists in the profession, nor is its study so difficult as to be at all impossible to the busy practitioner. He is the one who first meets with it in its early curable stage, and it should be alike his duty and his pleasure to instruct the public that the effects of alcohol penetrate to every part of the living body; that there are but few blood vessels; not a muscle; not an active part of any of the membranes; not a secreting gland, small or large; not an organ of sense; not a single viscus, whether pelvic, abdominal or thoracic, which is not interpenetrated by alcohol, when the alcohol habit be indulged in. It causes marked changes in the rate and force of the heart's action; in the tonicity of the muscular arteries; alters temperature; causes changes in rate of action of gland and muscles; causes changes in the varied forms of sensibility, from the most general to the most special, and the most grave changes of all in the most exalted of all functions comprised under the head of mind and mental operations, until, finally, the co-ordination function in the brain is overthrown and the consciousness of personal identity and responsibility are destroyed.

Alcoholic inebriety is a disease, and curable as other diseases are, and a very much larger per cent. of cases can be cured by regular systematic treatment than is now supposed. Merely keeping a patient away from liquor is the smallest and most unimportant part of treatment. The whole system, which is shattered and broken down, has to be invigorated and restored to its normal standard, and the brain and nervous system must be brought up to the highest resisting point, before the lost will-power is restored and the irresistible craving for alcohol cured. There is no disease of the nervous system more grave and more worthy of the most careful study and attention than dipsomania. It is a true periodic insanity, and only the most careful treatment, continued for months, can cure it. The treatment must be careful, methodical and intelligent, the physician recognizing the fact that he has to deal with a physical disease. If the profession will so regard it, and aim to instruct the communities in which they live as to the physiological effects of alcohol on the human body—that, primarily, it produces congestion from want of vaso-motor control, loss of muscular control, perversions of judgment and will-power, and absolute prostration of nerve power, while, secondarily, it causes hypertrophy of the heart, structural diseases of the stomach, brain, liver and kidneys; alcoholic dyspepsia; alcoholic phthisis and a multitude of nervous lesions which may end in alcoholic dementia, in which personal identity and responsibility are lost, and whose earliest symptoms are loss of memory, failure of speech and manifestations of moral obliquity, and finally delirium tremens, mania a potu, and perhaps chronic insanity. If the profession, we repeat, will so instruct their communities as to the action of alcohol on the functions of the human body, the science of preventive or state medicine will owe to such men an incalculable debt of gratitude for disease and death prevented and much domestic misery averted.

EDWARD C. MANN, M.D.

No. 128 Park Place, near Prospect Park, Brooklyn, N. Y.

